# Relationship between parental involvement and children’s positive mental character during early years: the moderating role of parent-teacher relationship and teacher-parent relationship

**DOI:** 10.3389/fpsyg.2024.1438784

**Published:** 2024-12-03

**Authors:** Liman Cai, Peishan Huang, Yuanfang Guo

**Affiliations:** ^1^Center for Studies of Psychological Application, South China Normal University, Guangzhou, China; ^2^School of Education, South China Normal University, Guangzhou, China; ^3^Faculty of Preschool Education and Humanities, Dongguan Polytechnic, Dongguan, China; ^4^Faculty of Education, University of Macau, Macau, China; ^5^Faculty of Child Development and Education, Zhejiang Normal University, Jinhua, China

**Keywords:** parental involvement, positive mental character, parent-teacher relationship, teacher-parent relationship, Chinese preschool children

## Abstract

**Introduction:**

To explore the association between parental involvement and children’s positive mental character, and examine the moderating effect of the relationship between teachers and parents.

**Methods:**

The present study conducted latent moderated structural equation model among 167 Chinese preschool teachers and 1019 preschool children and their parents. Parents reported children’s positive mental character and perceived relationships with their children’s teachers while teachers reported their perceived relationships with each child’s parents.

**Results:**

The results indicated that (1) three dimensions of parental involvement (home-based involvement, school-based involvement and home-school conferencing) were positively related to children’s positive mental character; (2) teacher-perceived teacher-parent relationship moderated the association between home-based involvement and children’s positive mental character; (3) the effects of school-based involvement * parent-teacher relationship and home-school conferencing * parent-teacher relationship on children’s positive mental character were significant.

**Discussion:**

This study provided the implications for promoting the development of positive mental character among Chinese preschool children.

## Introduction

Educational researchers have long been interested in the effect that parental involvement have on children’s development, such as academic achievement, social skills, socio-emotional aspect and mental health (Boonk et al., 2018; Castro et al., 2015; Cosso et al., 2022; Cripps and Zyromski, 2009; Nguyen et al., 2020; Nokali et al., 2010; Olvera and Olvera, 2012; Sheridan et al., 2019). Positive mental character refers to the relatively stable positive psychological traits gradually formed by the mutual influence of an individual’s intrinsic potential and environmental education (Seligman and Csikszentmihalyi, 2014). It is the core element that humans rely on for survival and development, an essential ability to overcome mental illnesses, and an important indicator of mental health (Seligman et al., 2005; Seligman and Csikszentmihalyi, 2014). Previous studies have demonstrated that parental involvement had a significantly impact on children’s positive mental character (Cripps and Zyromski, 2009; Dippel et al., 2022; Manassis et al., 2014; Thulin et al., 2014; Wang and Sheikh-Khalil, 2014).

Bronfenbrenner’s ecological theory indicates that the surrounding environment and interpersonal relationships could affect children’s psychological development, such as the relationship between parents and teachers (Bronfenbrenner, 1994). The relationship between parents and teachers interacted with parental involvement, which contributed to children’s positive mental character (Cook et al., 2018; Harpaz and Grinshtain, 2020; Hidayat and Arini, 2022). However, parent involvement includes three aspects: home-based involvement, school-based involvement and home-school conferencing (Fantuzzo et al., 2013; Liu and Li, 2019), and the relationship between parents and teachers also consists of two aspects: parent-perceived parent-teacher relationships and teacher-perceived teacher-parent relationships (Nzinga-Johnson et al., 2009). Due to the different perspectives on the quality of relationships, this study explored the effect of parental involvement and relationships between parents and teachers on children’s positive mental character from different perspectives.

### Parental involvement and children’s positive mental character

Consistent evidence has shown that parental involvement was associated with children’s positive mental character (Cripps and Zyromski, 2009; Dippel et al., 2022; Manassis et al., 2014; Payne, 2010; Thulin et al., 2014; Wang and Sheikh-Khalil, 2014). A meta-analysis study of 39 studies found that parental involvement had a positive and moderate effect on children’s overall performance including positive mental character (Cosso et al., 2022). Nokali et al. (2010) reached the similar conclusion after following elementary school students for 5 years. In a sample of 462 Chinese primary school students, more family involvement could increase children’s positive mental character regardless of family socioeconomic status (Nguyen et al., 2020). Meanwhile, other meta-analysis studies suggested that parental involvement is beneficial for the treatment of children’s mental health problems, more parental involvement accompanies children’s better mental health (Dippel et al., 2022; Manassis et al., 2014; Thulin et al., 2014).

Parent involvement is a multidimensional process that occurs across multiple contexts (Curtis et al., 2021). It includes home-based involvement, school-based involvement and home-school conferencing. It refers to the level of parents’ participation in children’s lives and studies (Fantuzzo et al., 2013; Liu and Li, 2019). Wang and Sheikh-Khalil (2014) found that parental involvement including its three dimensions predicted academic achievement and mental health both directly and indirectly through behavioral and emotional engagement among 1,056 American adolescent. A meta-analysis of 117 studies indicated the significant effects of home-school conferencing on children’s mental health (Sheridan et al., 2019). Limited studies have investigated the relation between three dimensions of parental involvement and children’s positive mental character. Olvera and Olvera (2012) indicated that home-school conferencing was associated with positive mental character among Latino children. Cosso et al. (2022) found that home-based and school-based parental involvement were not significantly associated with children’s positive mental character. However, Curtis et al. (2021) tracked 210 American children and found school-based parental involvement was related to children’s positive mental character significantly. It can be seen that scholars have not reached a consistent conclusion, and there were few studies that separately examined the relationship between the three dimensions of parental involvement and children’s positive mental character. Therefore, the present study explored this relationship among Chinese preschool children.

### The relationship between parent and teacher as a moderator variable

The collaboration between parents and teachers can promote children’s behavioral, academic, and mental health development (Cook et al., 2018; Eccles and Harold, 2013; Harpaz and Grinshtain, 2020; Hidayat and Arini, 2022; Iruka et al., 2011; Kim et al., 2013; Serpell and Mashburn, 2012). Especially in early childhood, better relationship between parent and teacher support child development across contexts (Lang et al., 2017). Smith et al. (2020) found that the relationship between parent and teacher positively influenced children’s mental health in a meta-analysis study including 77 parent-teacher partnership intervention studies. The higher level of cooperation between parents and teachers is beneficial for the development of children’s positive mental character. Lower quality parent-teacher relationships were significantly related to children’s mental health problems. Among 434 junior high school students in Indonesia, Anggraini and Sopianingsih (2023) found similar conclusion that better relationship between parent and teacher promoted children’s mental recovery, help them overcome psychological disorders such as depression and anxiety.

Previous studies have demonstrated that parental involvement was associated with the relationship between parent and teacher (An and Chang, 2021; Garbacz et al., 2016; Grace and Gerdes, 2019; Lang et al., 2017). A meta-analysis study found that parental involvement had significant effect on the relationship between parent and teacher across different culture, more engagement could promote relationships (Smith et al., 2022). A longitudinal study among Australian preschool children indicated that parental involvement predicted the relationship between parent and teacher across two waves (Murray et al., 2015). Other cross-sectional studies and qualitative studies have also reached the similar conclusion (An and Chang, 2021; Lang et al., 2017; Mirza, 2019). However, Nzinga-Johnson et al. (2009) found that both parent- and teacher-perceived parent-teacher relationships predicted parental involvement regardless of racial and socioeconomic factors in a sample of 483 American preschool children. Parental involvement interacted with the relationship between parent and teacher contributed to children’s academic achievement and mental health (Cook et al., 2018; Harpaz and Grinshtain, 2020; Hidayat and Arini, 2022). Therefore, in the process of parental involvement on children’s positive mental character, the relationship between parent and teacher may have a moderating role. Moreover, the relationship between parents and teachers includes two aspects: parent-perceived parent-teacher relationships and teacher-perceived teacher-parent relationships (Nzinga-Johnson et al., 2009). Due to different perspectives, there may be differences in these two relationships. This study explored the moderating effect of the relationship between parent and teacher from the different perspectives of both parents and teachers.

### The present study

Previous studies have indicated that parental involvement was related to children’s positive mental character (Dippel et al., 2022; Thulin et al., 2014; Wang and Sheikh-Khalil, 2014), but the effects of three dimensions of parental involvement on children’s positive mental character were not clear. The relationship between parent and teacher also played an important role in promoting children’s positive mental character (Anggraini and Sopianingsih, 2023; Lang et al., 2017; Smith et al., 2020). Parental involvement interacted with the relationship between parent and teacher had an impact on children’s positive mental character (Cook et al., 2018; Harpaz and Grinshtain, 2020; Hidayat and Arini, 2022). The relationship between parent and teacher might play a moderating role in the relationship. We also considered both parent and teacher reports of the relationship between parent and teacher given that these reports likely capture different aspects of and perceptions of the relationship. However, limited studies have explored this relationship among Chinese preschool children.

In China, most children aged 3 to 6 attend full-time preschool programs and stay with the teacher during three preschool years. They start preschool at age 3 and spend about 8 h per day in preschool (Guo et al., 2023; Wu et al., 2018). Chinese parents attach great attentions to the development of their children in all aspects, including academic performance, social skills, and positive mental character, and invest as much resources as possible to cultivate their children (Cui et al., 2024; Guo et al., 2023). Compared to other cultures, Chinese parents are highly involved in various aspects of their children’s development under the influence of Confucianism (Cui et al., 2024). To promote children’s development, parents actively maintain their relationship with teachers, and preschools also require teachers to communicate with parents timely and establish good teacher-parent relationships (Hu et al., 2021). Due to the high teacher-to-child ratio, parents and teachers may have different perspectives on the relationship between parent and teacher. Therefore, this study explored the relationship between three dimensions of parental involvement and children’s positive mental character, as well as the moderating role of parent-perceived parent-teacher relationships and teacher-perceived teacher-parent relationships separately in the Chinese context, which has important educational value and practical significance.

**Figure 1:**
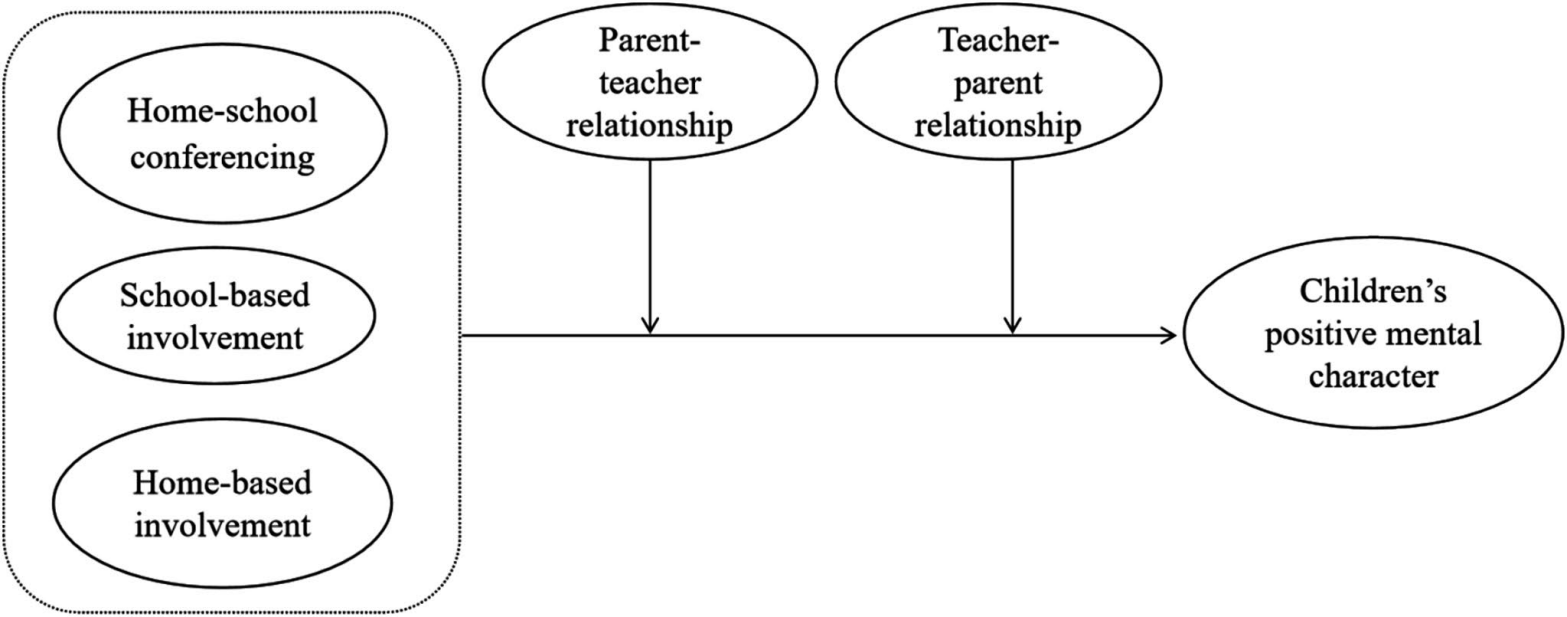
The hypothesized model.

The current research aims to (a) explore the relationship between three dimensions of parental involvement and children’s positive mental character; (b) identify the moderating role of parent-perceived parent-teacher relationships and teacher-perceived teacher-parent relationships in the relationship separately among Chinese preschool children. Accordingly, the following hypotheses were proposed, the hypothetical model was illustrated in Figure 1.

*Hypothesis 1*: Three dimensions of parental involvement were associated with Chinese preschool children’s positive mental character positively.

*Hypothesis 2*: Parent-perceived parent-teacher relationships and teacher-perceived teacher-parent relationships played a moderating role in the relationship.

## Methods

### Participants

The participants in this study were recruited in Guangdong province in southern China through a stratified sampling method. Guangdong province is China’s southern gateway to the world, representing China’s vast economic development and rapid modernization in culture and thought. Three regions representing low, medium, and high levels of economic development in 2022 in Guangdong province were identified; then, about 10–18 preschools in each region were randomly selected based on two main characteristics of preschools, namely, their funding source (public/private) and geographical location (urban/rural). A total of 38 kindergartens were selected. Then, we randomly selected classrooms serving 3-, 4-, 5-year old children in each preschool. Finally, about 8 preschoolers in each classroom were randomly recruited. By adopting this sampling procedure, we could ensure the representativeness of the children sample. Eventually, a total of 167 lead teachers and 1,019 parents in these classrooms agreed to participate in the study and signed informed consents, resulting to a total sample of 1,019 children. Both teachers and parents responded to an online questionnaire, respectively. Teacher reported their perceived relationship with each child’s parents while parents reported their perceived relationship with the child’s teacher and children’s positive character. This study underwent rigorous review by the institutional research ethics committee at the first authors’ university.

Table 1 presents demographic information about the participants. The recruited children sample showed a relatively equal distribution across different levels of economic development and grade levels of preschools. Male children accounted for 50.2% of the total. Of this sample, 63.2% came from public preschools, and 86.8% were from urban preschools. According to the China Education Statistical Yearbook (Ministry of Education of the People’s Republic of China, 2021), the composition of preschoolers in China is as follows: 54.2% of preschoolers come from public schools, and about 80% of preschoolers are from urban schools. Thus, this children sample can be regarded as representative of Chinese preschoolers. The average age of the teacher participants was 34.15 years old (SD = 6.76), with an average teaching experience of 12.83 years (SD = 8.10). The average age of the parent participants was 33.72 years old (SD = 4.59). The socioeconomic status (SES) of families was determined adhering to the methodology outlined by Ren (2010). Through a factor analysis, which encompassed three pivotal indicators: the peak educational attainment, occupational status, and yearly earnings of the children’s mothers, a singular factor emerged with an Eigenvalue exceeding unity, accounting for 63.60% of the total variance. Subsequently, the SES was quantified utilizing the formula: SES = (0.81 × mother’s education level + 0.72 × mother’s annual income + 0.85 × mother’s occupation) / 0.64.

**Table 1:** Demographics of participants.

	*N*	Mean	SD	Category	Frequency (Percentage)
Teacher age	169	34.15	6.76		
Teaching experience	169	12.83	8.10		
Parent age	1,019	33.72	4.59		
Family SES	1,019	13.02	5.21		
Region of preschools	38			High level of economic development	18 (47.4%)
				Medium level of economic development	10 (26.3%)
				Low level of economic development	10 (26.3%)
Geographical location of preschools	38			Urban	33 (86.8%)
				Rural	5 (13.2%)
Funding sources of preschools	38			Public	24 (63.2%)
				Private	14 (36.8%)
Children gender	1,019			Boys	512 (50.2%)
				Girls	507 (49.8%)
Children grade	1,019			Junior class	338 (33.2%)
				Middle class	364 (35.7%)
				Senior class	317 (31.1%)

SES, socioeconomic status.

### Measures

#### Parental involvement

The 20-item Family Involvement Questionnaire-Short Form (FIQ-SF) validated by Liu and Li (2019) in the Chinese context was used to assess parents’ involvement and participation in their children’s academic and life experiences. This scale, originally developed by Fantuzzo et al. (2013), is a widely used measure to assess family participation in early childhood education. It covers three dimensions, namely home-school conferencing (7 items; e.g., *“1.I attend conferences with the teacher to talk about my child’s learning or behavior.”*), school-based involvement (6 items; e.g., *“I participate in planning classroom activities with the teacher.”*), and home-based involvement (7 items; e.g., *“I talk about my child’s learning efforts in front of relatives and friends.”*). Each strategy was rated on a 5-point Likert-type scale ranging from 1 (never) to 5 (always). Higher total score of all items represents parents’ higher level of engagement in children’s academic and life success. In our study, the Cronbach’s alpha coefficients of home-school conferencing, school-based involvement, and home-based involvement were 0.91, 0.88, and 0.85, respectively. Using Confirmatory factor analysis (CFA), the three-factor model of this scale showed an acceptable model fit (*χ^2^* = 1313.97, *df* = 167, CFI = 0.91, TLI = 0.89, RMSEA = 0.08, SRMR = 0.06). It is notable to mention that, although TLI value for this model was slightly lower than the recommended value, it should not be the sole basis for rejecting the overall model fit, particularly when considering that other fit indices all indicated good model fit (Maruyama, 1998).

#### Teacher-parent relationship

Teachers’ perceived relationship with each child’s parent was measured by using a 7-item Home-school Relationship Scale which was developed by Barbarin (2000a) for the National Center for Early Development and Learning (NCEDL) Pre-K study program. The scale asked teachers to report their overall satisfaction with interactions with the child’s parent (i.e., *“How would you describe your relationship and interactions with this child’s parents?”*), the emotional tone of the relationship, degree of trust, clarity of communication, agreement about issues affecting the child, feelings of being appreciated by the child’s parent, and degree of support and cooperation. Each item was measured on a 4-point Likert scale from very negative to very positive. Higher scores of all items indicate teachers perceived a more positive relationship with the parent. In our study, Cronbach’s alpha coefficient of this scale was 0.90. The one-factor CFA model of this scale showed a good model fit (*χ^2^* = 73.86, *df* = 10, CFI = 0.98, TLI = 0.97, RMSEA = 0.08, SRMR = 0.02).

#### Parent-teacher relationship

Relationship with Child’s School Scale, also developed by Barbarin (2000b) for the NCEDL Pre-K study program, was used to measure parents’ perceptions of relationship with their child’s teacher. The scale has five items, including the frequency of contact with the teacher (i.e., *“How often do you talk with your child’s teacher either on the phone or in person?”*), the degree of trust and agreement with the teacher, clarity of communication, and overall satisfaction. The original version of this scale used either a 4-point or 5-point scoring system. However, to enhance participants’ readability and ensure consistency, each item has been slightly adjusted to a 5-point scale, ranging from very negative to very positive. Higher scores of all items indicate parents perceived a more positive relationship with the child’s teacher. In our study, Cronbach’s alpha coefficient of this scale was 0.70. The one-factor CFA model of this scale showed a good model fit (*χ^2^* = 7.07, *df* = 5, CFI = 0.99, TLI = 0.99, RMSEA = 0.02, SRMR = 0.01).

#### Children positive mental character

Parents completed a 37-item scale named as Preschool Children’s Positive Mental Character Questionnaire (PCPMCQ, Han et al., 2019). This scale covers five dimensions, namely, wisdom (10 items; e.g., *“The child often has a* var*iety of novel ideas.”*), courage (5 items; e.g., *“The child lacks patience when studying or playing.”*), benevolence (10 items; e.g., *“The child is willing to share their things (such as snacks, toys,* etc.*) with others.”*), justice (6 items; e.g., *“When seeing other children being bullied, the child always feels indignant.”*), and restraint (6 items; e.g., *“When sustaining minor injuries (such as falling down or bumping into something), the child can still keep calm.”*). Each dimension was measured on a 5-point Likert scale from 1 (strongly disagree) to 5 (strongly agree). Items were summed so that high scores indicated a higher level of positive mental character. In our study, Cronbach’s alpha coefficient of this scale was 0.90. For the model’s simplicity, the CFA model of this scale was conducted through item parceling, showing a good model fit (*χ^2^* = 11.50, *df* = 5, CFI = 0.99, TLI = 0.97, RMSEA = 0.05, SRMR = 0.02).

### Analytical strategy

Statistical analyses were performed with three steps in this research. First, descriptive statistics, normality tests, and correlation analysis of the variables were performed with SPSS 27.0. The reliability coefficients of each measurement were also calculated using SPSS 27.0. Second, in order to estimate the construct validity of each scale in this study, three confirmatory factor models were built separately for parental involvement, parent-teacher relationship, teacher-parent relationship, and children positive mental character using Mplus 8.0. Third, the hypothesized model, including the main effects of parental involvement and the moderation effects of teacher-parent relationship and parent-teacher relationship in the associations between parental involvement and children’s positive mental character, were estimated by latent moderated structural equation (LMS) (Klein and Moosbrugger, 2000). Compared to traditional regression analysis, utilizing LMS to examine the moderating effect can better estimate measurement errors (Edwards and Lambert, 2007). In addition, LMS does not require interaction terms to follow a normal distribution, thus avoiding estimation biases that arise when the interaction terms do not adhere to normality assumptions (Moosbrugger et al., 2009). Since the three latent factors of parental involvement (i.e., home-based involvement, school-based involvement, and home-school communication) reflected different aspects, three LMS models were built separately for each moderator. A significant interaction effect indicated that the association between parental involvement and children’s positive mental character changed as a function of the two moderator variables. The follow-up analyses of the significant interaction were conducted by separately calculating and visualizing the simple slopes for low (−1 SD) and high (+1 SD) levels of teacher-parent relationship and parent-teacher relationship.

In the current study, several indices were used to assess the model fit, including the Chi-square statistic (*χ*^2^), root mean square error of approximation (RMSEA), Tucker-Lewis index (TLI), comparative fit index (CFI) and the standardized root mean square residual (SRMR). Following the recommended criteria, CFI > 0.90, TLI > 0.90, RMSEA <0.08 and SRMR <0.08 were used as cutoffs to indicate an acceptable data fit (Schreiber et al., 2006). Besides, since LMS does not provide these model fit indices, an initial model without interaction terms or algorithms was designed to determine the model fit firstly. Next, a full model with the integration algorithm was examined. A chi square difference test was then used to compare the −2 log likelihood (−2LL) values of these two models. If the initial model fits well and the −2LL value test is significant, it can be considered that the LMS model also fits well (Raufelder et al., 2018). Additional demographic variables were included as covariates in the models if they were significantly related to children’s positive mental character.

## Results

### Common method bias

Since this study primarily relied on self-reported data, it is crucial to examine common method bias in order to ensure the reliability of the collected data. Harman’s single-factor method is an effective and widely used technique for detecting substantive common method effects (Aguirre-Urreta and Hu, 2019). The results revealed that 11 factors had an Eigenvalue greater than 1, and the variance explained by the first factor was 21.61%, which is below the critical threshold of 40%. Consequently, no significant common method bias was observed in this study.

### Descriptive statistics

Table 2 shows the mean and standard deviation, skewness and kurtosis of the variables under study. Based on the skewness and kurtosis value, the data in this research were found to be normally distributed. Among the three aspects of parental involvement, the parents reported the highest scores on home-based involvement (*M* = 3.38, SD = 0.63), followed by school-based involvement (*M* = 3.21, SD = 0.77), and the lowest scores were reported for home-school conferencing (*M* = 3.12, SD = 0.70). Both parents’ perceptions of the relationship with teachers (*M* = 3.55, SD = 0.44) and teachers’ perceptions of the relationship with parents (*M* = 4.23, SD = 0.49) were positive. Children’s positive mental character reported by their parents was at the upper-middle level (*M* = 3.70, SD = 0.41).

**Table 2:** Descriptive statistics and correlations.

	1	2	3	4	5	6	7	8	9	Cronbach’s alpha
1.Children grade	–									–
2.Children gender	−0.02	–								–
3.Family SES	−0.05	−0.01	–							–
4.Home-based involvement	0.001	−0.04	0.23^**^	–						0.85
5.School-based involvement	0.02	−0.02	0.14^**^	0.67^**^	–					0.88
6.Home-school conferencing	−0.01	−0.04	0.12^**^	0.72^**^	0.73^**^	–				0.91
7.Teacher-parent relationship	0.08^*^	−0.03	0.09^**^	0.06	0.06	0.07^*^	–			0.90
8.Parent-teacher relationship	−0.02	−0.03	0.03	0.28^**^	0.32^**^	0.44^**^	0.05	–		0.70
9.Children’s positive mental character	0.04	0.03	0.10^**^	0.50^**^	0.36^**^	0.40^**^	0.09^**^	0.30^**^	–	0.90
Skewness	0.04	0.01	0.22	0.41	0.16	0.44	−0.78	−1.39	0.26	–
Kurtosis	−1.44	−2.0	−0.93	0.23	−0.27	0.36	0.17	2.27	0.30	–
Mean	–	–	13.02	3.38	3.21	3.12	3.55	4.23	3.70	–
Standard deviation	–	–	5.21	0.63	0.77	0.70	0.44	0.49	0.41	–

*p < 0.05, **p < 0.01. Gender was treated as a dummy variable where males are represented by 1 and females by 2. Children grade was treated as a dummy variable where children from junior class represented by 1, those from middle class by 2, and those from senior class by 3.

SES, socioeconomic status.

### Correlations

Table 2 presents inter-correlations among the variables of interest. The results showed that most of variables were significantly correlated with each other, except for the relationships between the two aspects of parental involvement (i.e., home-based involvement, school-based involvement) and teacher-parent relationship. Specifically, all the three aspects of parental involvement significantly related to children’s positive mental character (home-based involvement: *r* = 0.56, *p* < 0.01; school-based involvement: *r* = 0.36, *p* < 0.01; home-school conferencing: *r* = 0.40, *p* < 0.01). The two potential moderators were also found to be significantly related to the children’s outcome variable (teacher-parent relationship: *r* = 0.09, *p* < 0.01; parent-teacher relationship: *r* = 0.30, *p* < 0.01). Children’s grade, gender, and family SES were proposed as potential covariates; however, only family SES was significantly related to the children’s outcome (*r* = 0.10, *p* < 0.01).

### Measurement models

Before testing the moderation models, CFA was used to examine if measurement models fit well. Results showed the whole measurement model including parental involvement, teacher-parent relationship and children’s positive character reached an acceptable model fit (*χ^2^* = 2210.77, *df* = 454, *p* < 0.001, CFI = 0.90, TLI = 0.90, RMSEA = 0.06, SRMR = 0.05). The measurement model for parent-teacher relationship also showed an acceptable model fit (*χ^2^* = 2163.82, *df* = 395, *p* < 0.001, CFI = 0.89, TLI = 0.87, RMSEA = 0.07, SRMR = 0.07).

### Latent moderated models

To test the moderating effects of teacher-parent relationship (see Table 3, model 1 to model 3) and parent-teacher relationship (model 4 to model 6), in the link between three aspects of parental involvement and children’s positive character, a total of 6 LMS models were performed, respectively. Family SES was taken as a control variable in these models since it significantly related to children’s positive character.

**Table 3:** Model comparisons and model fit indices for all LMS models.

Model fit indices for baseline models							Model comparisons between baseline models and LMS models					
	*χ^2^*	*df*	CFI	TLI	RMSEA	SRMR	Log likelihood	AIC	Free parameters	−2 log likelihood (−2LL)	*Δ*AIC	Results
Teacher-parent relationship as the moderator												
Model 1-1: HI + TPR + PMC	775.44	149	0.93	0.92	0.06	0.04	−15466.694	31053.389	60	*p* < 0.05	2.26	↑
Model 1-2							−15463.567	31051.133	62			
Model 2-1: SI + TPR + PMC	744.06	132	0.93	0.92	0.07	0.04	−14597.261	29308.522	57	*p* > 0.05	−1.66	↓
Model 2-2							−14596.089	29310.178	59			
Model 3-1: HS + TPR + PMC	715.73	149	0.94	0.93	0.06	0.03	−14835.024	29790.049	60	*p* > 0.05	−2.19	↓
Model 3-2							−14834.122	29792.243	62			
Parent-teacher relationship as the moderator												
Model 4-1: HI + PTR + PMC	506.32	116	0.93	0.91	0.06	0.05	−16669.347	33446.693	54	*p* < 0.05	1345.29	↑
Model 4-2							−15994.704	32101.408	56			
Model 5-1: SI + PTR + PMC	503.10	101	0.93	0.92	0.06	0.06	−15791.274	31684.549	51	*p* < 0.05	4.11	↑
Model 5-2							−15787.220	31680.441	53			
Model 6-1: HS + PTR + PMC	628.56	116	0.93	0.91	0.07	0.07	−16003.895	32115.789	54	*p* < 0.001	14.38	↑
Model 6-2							−15994.704	32101.408	56			

HI, home-based involvement; SI, school-based involvement; HS, home-school conferencing; TPR, teacher-parent relationship, PTR, parent-teacher relationship; PMC, positive mental character; AIC, Akaike information criterion; Model 1-1, 2-1, 3-1, 4-1, 5-1, and 6-1 represents baseline models (models without interaction terms), while model 1-2, 2-2, 3-2, 4-2, 5-2, and 6-2 represents LMS models (models including interaction terms); Family SES has been included as the covariate in all LMS models; ↑ signifies the LMS model outperforms the baseline one, while ↓ indicates it performs worse.

#### Teacher-parent relationship as the moderator

**Figure 2:**
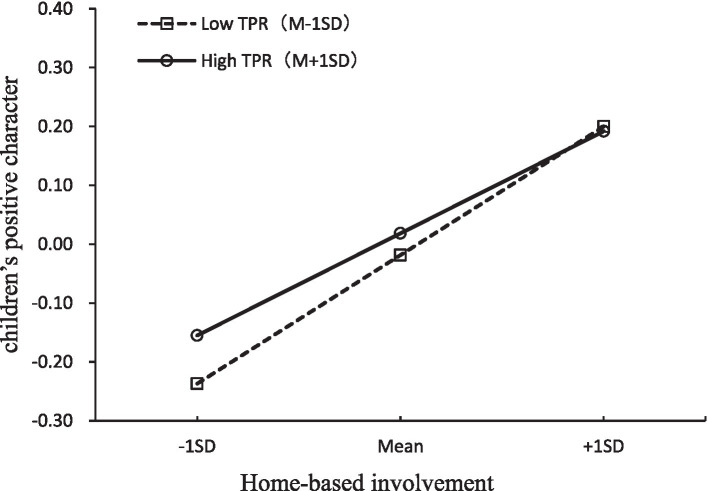
Teacher-parent relationship as a moderator of the associations between home-based involvement and children’s positive character. TPR, teacher-parent relationship.

First, we examined the moderating role of teacher-parent relationship in the associations between home-based involvement and children’s positive character. A baseline model solely including the main effect of home-based involvement (without interaction terms) was initially built (model 1-1), which showed a good model (*χ^2^* = 775.44, *df* = 149, CFI = 0.93, TLI = 0.92, RMSEA = 0.06, SRMR = 0.04). A model with interaction terms teacher-parent relationship * home-based involvement (model 1-2) was then built. As indicated in Table 3, the chi square difference test for −2LL values between model 1-1 and 1-2 yielded a significant result (*p* < 0.05). Moreover, the AIC value for model 1-1 was higher than that for model 1-2 (*Δ*AIC = 2.26). Based on these findings, the LMS model (model 1-2) for home-based involvement could be considered as better than the baseline model (model 1-1). The results revealed that the main effects of home-based involvement on children’s positive character was significant (*β* = 0.40, *p* < 0.001) while those for teacher-parent relationship was not significant (*β* = 0.04, *p* > 0.05). The interaction between teacher-parent relationship and home-based involvement was a statistically significant predictor of children’s positive character (*β* = −0.11, *p* < 0.05). Specifically, as Figure 2 showed, for children with weaker teacher-parent relationships, a higher level of home-based involvement was strongly associated with greater development in positive character (*β* = 0.51, *p* < 0.001), whereas for those with higher levels of teacher-parent relationship, the effects of home-based involvement on children outcome was attenuated (*β* = 0.29, *p* < 0.001). This suggested that the impacts of parents’ involvement in family activities on fostering children’s positive character development may be particularly pronounced among those with weaker teacher-perceived relationships with parents.

Second, we examined the moderating role of teacher-parent relationship in the associations between school-based involvement and children’s positive character. The main effect of school-based involvement on children’s positive character was significant (*β* = 0.16, *p* < 0.001). However, based on the results of model 2-1 and 2-2 in the Table 3, this LMS model did not fit well, and the interaction term teacher-parent relationship * school-based involvement was not significant (*β* = −0.05, *p* > 0.05). Finally, the moderating effects of teacher-parenting relationship in the associations between home-school conferencing and children’s positive character were also examined. The main effects of home-school conferencing on children’s positive character was significant (*β* = 0.23, *p* < 0.001). However, based on the results of model 3-1 and 3-2 in the Table 3, this LMS model did not fit well, and the interaction term teacher-parent relationship * home-school conferencing was not significant (*β* = −0.05, *p* > 0.05). The results for school-based involvement and home-school conferencing indicated that their positive associations with children’s positive character remained consistent, unaffected by being embedded in varying relational contexts.

#### Parent-teacher relationship as the moderator

First, the main effects of home-based involvement (*β* = 0.36, *p* < 0.001) and parent-teacher relationship (*β* = 0.20, *p* < 0.001) were significant in predicting children’s positive character. However, the interaction term parent-teacher relationship * home-based involvement was not statistically significant (*β* = 0.15, *p* = 0.07), although this LMS model (model 4-2) was better than its baseline model (model 4-1). This result suggested that the combined influence of parents’ home-based involvement and their relationship with teachers did not exhibit a statistically significant multiplicative effect on children’s positive character.

**Figure 3:**
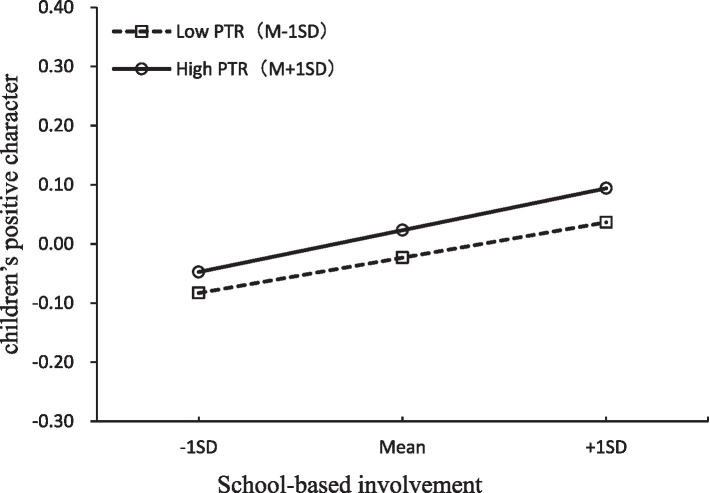
Parent-teacher relationship as a moderator of the associations between school-based involvement and children’s positive character. PTR, parent-teacher relationship.

Second, based on the results of model 5-1 and 5-2 in the Table 3, the LMS model with interaction term school-based involvement * parent-teacher relationship fit well. The main effects of school-based involvement (*β* = 0.13, *p* < 0.001) and parent-teacher relationship (*β* = 0.26, *p* < 0.001) were both significant, while their interaction term was marginally significant (*β* = 0.12, *p* = 0.05). Specifically, as Figure 3 showed, for children with higher levels of parent-teacher relationship, a higher level of school-based involvement was strongly associated with children’s greater development in positive character (*β* = 0.25, *p* < 0.001), whereas for children with lower levels of parent-teacher relationship, the associations between school-based involvement and children outcome did not change as a function of parent-teacher relationship (*β* = 0.008, *p* > 0.05). This indicated that the effectiveness of school-based involvement in promoting positive character development in children was contingent upon the quality of the parent-teacher relationship. When this relationship was strong, increased school-based involvement had a substantially more profound and positive impact on children’s character development.

**Figure 4:**
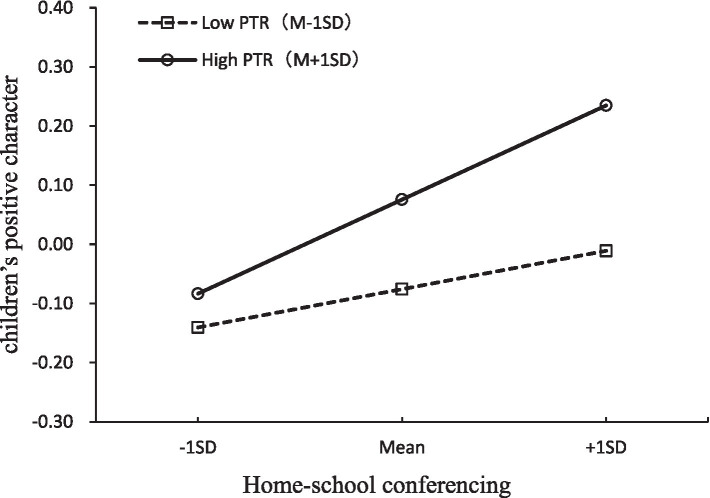
Parent-teacher relationship as a moderator of the associations between home-school conferencing and children’s positive character. PTR, parent-teacher relationship.

Finally, as demonstrated in the results of model 6-1 and 6-2 in the Table 3, the tests regarding the LMS model with interaction term home-school conferencing * parent-teacher relationship exhibited a good fit with the data. The main effects of home-school conferencing (*β* = 0.18, *p* < 0.001) and parent-teacher relationship (*β* = 0.21, *p* < 0.001), as well as their interaction term (*β* = 0.20, *p* < 0.001) were all statistically significant. Specifically, as Figure 4 showed, for children with higher levels of parent-teacher relationship, a higher level of home-school conferencing was strongly associated with children’s greater development in positive character (*β* = 0.38, *p* < 0.001), whereas for children with lower levels of parent-teacher relationship, the associations between home-school conferencing and children outcome did not change as a function of parent-teacher relationship (*β* = −0.03, *p* > 0.05). This underscored the critical role of regular and effective communication between homes and schools, particularly when coupled with strong parent-teacher relationships, in fostering significant improvements in children’s positive character development.

## Discussion

The present study explored the association between three dimensions of parental involvement and children’s positive mental character, and examined the moderating effect of parent-perceived parent-teacher relationships and teacher-perceived teacher-parent relationships in the relationship. The study found that all three dimensions of parental involvement (home-based involvement, school-based involvement and home-school conferencing) were positively related to children’s positive mental character. By using LMS to examine the moderating effect, this study indicated that teacher-perceived teacher-parent relationship moderated the association between home-based involvement and children’s positive mental character. The effects of teacher-parent relationship * school-based involvement and teacher-parent relationship * home-school conferencing on children’s positive mental character were not significant. Meanwhile, parent-perceived parent-teacher relationship served as a moderator, the effects of school-based involvement * parent-teacher relationship and home-school conferencing * parent-teacher relationship on children’s positive mental character were significant.

### The relationship between parental involvement and positive mental character

This study found that parental involvement including home-based involvement, school-based involvement and home-school conferencing was positively related to children’s positive mental character. This result was consistent with previous studies, the more parents’ involvement, the higher level of positive mental character children possesses (Cosso et al., 2022; Dippel et al., 2022; Nguyen et al., 2020; Thulin et al., 2014; Wang and Sheikh-Khalil, 2014). Preschool children, aged 3 to 6 years old, lack the ability to take care of themselves and make basic judgments, which rely on their parents’ education and guidance (Guo et al., 2023). According to the attachment theory (Bowlby and Ainsworth, 2013), parents are involved more in children’s lives and studies, creating a warm and safe atmosphere, which is beneficial for developing children’s positive mental character.

Positive mental character includes wisdom, courage, kindness, and so on, all these good qualities require parents to spend more energy and time cultivating young children (Han et al., 2019). For example, Parents often care and accompany their children, regularly communicate with teachers, and actively participate in preschool activities. During these processes, parents educate children on how to interact with others, how to deal with problems in life, and helping them learn to view the world around them correctly, these are all conducive to the formation and development of children’s positive mental character. Especially in the Chinese context, under the influence of Confucian, children face great pressure from an early age and need to learn various skills such as dancing, musical instruments, art, weiqi, and so on in extracurricular classes. The participation and guidance of parents at home and school, as well as active communication and cooperation with teachers, can help them form positive mental character in the learning process.

### Teacher-parent relationship as the moderator

Based on the attachment theory (Bowlby and Ainsworth, 2013), parental involvement plays a key role in children’s mental health. When children trust their parents and regard them as a secure base, they can explore the surrounding environment actively, play and work on their own, it is beneficial for the development of positive mental character (Cosso et al., 2022; Dippel et al., 2022; Manassis et al., 2014; Nguyen et al., 2020; Thulin et al., 2014; Wang and Sheikh-Khalil, 2014). Parents take care of their children’s lives and participate in their learning at home, such as accompanying them in reading and practicing, as well as taking them on trips and visiting museums, which is beneficial for children to develop positive mental character.

In the Chinese context, the principle of parenting has undergone significant changes in the past 40 years due to the implementation of the family planning policy. Compared to other cultures, Chinese parents pay more attention to their children, including various aspects such as life and study (Guo et al., 2023). Unlike the parent-teacher relationship reported by parents, teachers communicate and cooperate with the parents of all children in their responsible class. When the teacher reports a high level of teacher-parent relationship, it means that parents are overly concerned about their children, frequently contacting the teacher in an attempt to know everything about their children in the preschool, and spending a lot of time and energy communicating with the teacher to make sure their children receive good care in the preschool (Barbarin, 2000a). It can be seen that parents not only have a high level of participation in family life, but also excessive worry about their children’s preschool life, which is not conducive to the development of children’s positive mental character, which is consistent with existing research results from other age groups (Bradley et al., 2013; Cui et al., 2024; Gan and Bilige, 2019; Liou et al., 2019). On the contrary, when teachers report low-level teacher-parent relationships, parents focus on participating in children’s family life, which is more conducive to cultivating children’s positive mental character, consistent with research results from other age groups (Cui et al., 2024; Pomerantz et al., 2007; Kim, 2020; Wei, 2012).

### Parent-teacher relationship as the moderator

This study indicated that the higher level of parent-teacher relationship interacted with higher level of school-based involvement and home-school conferencing had a significantly effect on children’s positive mental character. Different from the teacher-parent relationship reported by teachers, parents only face preschool teachers who are in charge of their children’s class. Under the influence of Confucianism, Chinese parents respect and trust teachers, recognize their work, and actively cooperate with their work (Guo et al., 2023; Hu et al., 2021). Parents report more school-based involvement and home-school conferencing, it means that they actively participate in parent–child activities in preschools, timely communicate with teachers about their children’s situation, discuss appropriate educational strategies for promoting children’s positive mental character.

However, based on attachment theory (Bowlby and Ainsworth, 2013), parents are very important others for children, and play a crucial role in their overall development. Especially for preschool children, their independence is developing and they lack the ability to take care of themselves. They rely heavily on their parents in both live and studies, and parents’ influence on their psychological development is enormous (Guo et al., 2023; Wu et al., 2018). Therefore, for children with parents who actively participate in preschool activities and keep communication with teachers, creating a good learning and living environment for children could promote the development of children’s positive mental character, regardless of the lower level of parent-teacher relationships.

## Conclusion, limitations and implications

This study explored the relationship between three dimensions of parental involvement and children’s positive mental character, and the moderating role of parent-perceived parent-teacher relationships and teacher-perceived teacher-parent relationships separately among Chinese preschool children. The results indicated that three dimensions of parental involvement (home-based involvement, school-based involvement and home-school conferencing) were positively related to Chinese preschool children’s positive mental character. Teacher-perceived teacher-parent relationship moderated the relationship between home-based involvement and children’s positive mental character. Meanwhile, the effects of school-based involvement * parent-teacher relationship and home-school conferencing * parent-teacher relationship on children’s positive mental character were significant.

There are several limitations that warrant discussion. First, all participants were from Guangdong province in mainland China, which may not be representative of the whole country. Future research should expand the scope of sample recruitment to make it more generalizable. Second, the study examined the moderation models using the cross-sectional design. Further research should collect longitudinal data to confirm the causal relationship between parental involvement and children’s positive mental character. Third, all data in this study were collected by self-reported questionnaire, which may lead to overestimations of correlations among factors due to common method variance and a subjective social desirability bias (Podsakoff et al., 2012). Other assessment methods should be used in the future studies, such as observation and interviews could be considered. Lastly, this study compared latent moderated structural equation model and provided evidence about the relation between parental involvement and children’s positive mental character in the Chinese context. Future studies should conduct this research design in another cultural context.

The present study expands the application of attachment theory in early education and also has significant implications. For children’s positive mental character development, parents should increase their home-based involvement, such as spending more time accompanying children in learning, visiting and playing outside. Meanwhile, parents should actively participate in parent–child activities in preschools, maintain communication with teachers to timely understand children’s development, and cooperate with teachers well. Moreover, parents should establish a good parent-teacher relationship, trust teachers, regularly communicate and exchange ideas, and jointly promote the children’s development of positive mental character. It is very important for parents to believe that teachers could take good care of young children and not be overly anxious or sensitive, as this is not conducive to the development of children’s positive mental character.

## Data Availability

The raw data supporting the conclusions of this article will be made available by the authors, without undue reservation.
